# Outcome of Hypofractionated Palliative Radiotherapy Regimens for Patients With Advanced Head and Neck Cancer in Tikur Anbessa Hospital, Ethiopia: A Prospective Cohort Study

**DOI:** 10.1200/GO.23.00253

**Published:** 2024-01-05

**Authors:** Girum Tessema Zingeta, Yohannes Tesfaye Worku, Munir Awol, Edom Seife Woldetsadik, Mathewos Assefa, Tsion Zebdios Chama, Jilcha Diribi Feyisa, Hawi Furgassa Bedada, Mohammed Ibrahim Adem, Tariku Mengesha, Rebecca Wong

**Affiliations:** ^1^ Department of Oncology, School of Medicine, Addis Ababa University, Addis Ababa, Ethiopia; ^2^ Department of Radiation Oncology and Applied Sciences, Dartmouth Cancer Center, Lebanon, NH; ^3^ Department of Oncology, Saint Paul Hospital Millennium Medical College, Addis Ababa, Ethiopia; ^4^ St Peter Specialized Hospital, Addis Ababa, Ethiopia; ^5^ Princess Margaret Cancer Centre, University of Toronto, Toronto, ON, Canada

## Abstract

**PURPOSE:**

Head and neck cancers (HNCs) are the third most commonly treated cancer with radiation in Ethiopia. Most patients present with advanced stage and are not candidates for curative treatment. The objective of our study is to assess the outcome of hypofractionated palliative radiotherapy (RT) for advanced HNCs in a resource-limited setting.

**MATERIALS AND METHODS:**

Patients with histology-proven advanced HNC candidates for hypofractionated palliative RT were enrolled. Three regimens were allowed: 44.4 Gy in 12 fractions, 30 Gy in 10 fractions, and 20 Gy in five fractions. Response to treatment was assessed at baseline and at 4 weeks after treatment completion. The Kaplan-Meier curve was used to measure the survival.

**RESULTS:**

Between January 2022 and January 2023, 52 patients were enrolled and 25 patients were eligible for outcome assessment. Index symptoms include pain, bleeding, dysphagia, respiratory distress, and others in 25, 13, 10, 6, and 17 patients, respectively. Complete relief of the top three symptoms include pain in 52% of patients, hemostasis in 84% of patients, and dysphagia in 30% of patients. Objectively, 64% of patients attained partial response. For 48% of patients, their quality of life (QoL) improved in one parameter of the physical scores. Moreover, 64% of patients showed improvement in three parameters. The global functional score improved in 80% of patients. One patient had grade 3 xerostomia. At the end of the study period, 44% of patients died. The median survival after radiation was 9 months (95% CI, 7.2 to 10.8)

**CONCLUSION:**

All palliative hypofractionated regimens used were effective in terms of symptom control, tumor response rate, and QoL, and were well tolerated. This makes it appropriate for our setup because the majority of patients require palliation.

## Teaser

Palliative radiotherapy regimens have improved outcome of patients with advanced head and neck cancer in Ethiopia.

## INTRODUCTION

Head and neck cancers (HNCs) account for approximately 900,000 cases and over 400,000 deaths worldwide each year.^1^ HNCs are common in many parts of the world where tobacco and alcohol use is prevalent.^2^

**CONTEXT**

**Key Objective**
In resource-limited setups, what is the outcome of hypofractionated palliative radiotherapy (RT) regimens for patients with advanced head and neck cancer?
**Knowledge Generated**
The majority of the patients achieved symptom palliation and improved quality of life. Only one patient had grade 3 xerostomia.
**Relevance**
In Ethiopia, it is essential to provide hypofractionated palliative RT for symptom palliation when curative treatment is not amenable.

The global risk and mortality of HNCs are high in developing countries, which account for 82% of all HNC-related deaths and 67% of their volume globally.^3^ There are many challenges in Africa to improve the outcome of patients with HNCs.^4^ Although there is no national cancer registry in Ethiopia, studies suggest HNCs account for the third most commonly treated cancer with radiation next to gynecologic and breast cancers.^5^

The majority of HNC cases worldwide in high-risk areas are at an advanced, incurable stage.^6^ Patients who are not candidates for radical curative-intent therapy may still be candidates for palliative cancer-directed radiotherapy (RT).^7^ The goal of palliative care is to alleviate symptoms caused by cancer while minimizing treatment toxicity and side effects.^7^ In Ethiopia, most patients present at an advanced stage where curative treatment strategies are not amenable.^8^

In general, there is a paucity of research on palliative care, with significant gaps in the literature relating to dose and fractionation, identifying patients who are suited for palliation, the degree or duration of symptom reduction, and treatment-related toxicities. Furthermore, because of resource constraints, a long RT waiting list, and the poor socioeconomic status of patients in Ethiopia, shorter palliative RT regimens are an ideal choice for our setup if found feasible. This research aims to assess the outcome of hypofractionated palliative RT for advanced HNCs in a resource-limited setup.

## MATERIALS AND METHODS

### Study Design

The study is an unmatched prospective cohort study conducted at Tikur Anbessa Specialized Hospital (TASH) adult oncology department, which provides RT, chemotherapy, and palliative treatment to patients with solid cancer. The study period was from January 2022 to January 2023.

Patients with biopsy-confirmed HNCs having adequate baseline clinical assessment and imaging with advanced-stage disease not amenable to curative treatment were included. We excluded patients who had received RT before, patients with ambiguous diagnoses and unknown primary HNCs, and patients outside of the study period.

### Ethics Approval

Ethical approval was sought from the Addis Ababa University, School of Medicine, Department of Oncology (Ref No: 001/23/12/2021). Confidentiality and anonymity of the study were kept. Study participants were involved voluntarily and written informed consent was obtained from each participant after explaining the purpose of the study.

### Data Collection Procedure

The treating oncologist examined patients before the start of each course of RT, which included detailed history, physical examination, complete cell count, renal and liver function tests. Appropriate imaging for the tumor site staging with head and neck magnetic resonance imaging or computed tomography (CT) scan and metastatic workup with chest X-ray (CXR) and abdominal ultrasound were implemented. A CT scan confirmed any ambiguous lesion on CXR or abdominal ultrasound. The patient's tumor stage, performance status, as well as sociodemographic characteristics were documented during the initial evaluation. Pretreatment symptoms of bleeding, discharge from the tumor, dysphagia, pain, voice change, respiratory distress, and cough were also recorded.

After 4 weeks of treatment completion, response to treatment was measured according to the patient index symptoms (scored based on a four-point categorical scale: none, mild, moderate, and severe), and a clinical assessment of response to the primary tumor was based on direct inspection and palpation, as well as imaging using RECIST criteria. The Common Terminology Criteria for Adverse Events (CTCAE) were used to evaluate treatment toxicity. To better capture acute toxicities, toxicities were assessed on the last day of RT and after 1 month.

Direct patient interviews and secondary data from patient charts as well as electronic medical records were used as a data source. Variables related to HNC symptoms were selected in the European Organisation for Research and Treatment of Cancer Quality of Life Questionnaire (EORTC QLQ-C30) and EORTC QLQ-HN.43 to assess the quality of life (QoL) before treatment initiation and 4 weeks after treatment. We used the Amharic version of EORTC-QLQ 30/HN.43 validated tools and the reliability of the tools were checked by using Cronbach alpha value (α = .752). We assessed toxicity objectively using CTCAE version 5, because of which no Amharic translation were required. Overall survival time were assessed from the initial date of diagnosis to the date of death. To see the outcome (death or alive), patients were followed every month with a phone call. Time to death after completion of RT was also assessed.

Each patient chart was assigned a code number before data collection. Data were collected by two trained oncology residents. Before the actual study, the data extraction format was pretested and appropriate changes were made.

### Operational Definitions

#### Symptom Response

Complete response (CR) was defined as total symptom resolution; partial response (PR) as some symptomatic improvement; stable disease as no symptomatic improvement; and progressing disease as symptom worsening.^9^

#### RECIST Version 1.1

A standard way to measure how well a patient with cancer responds to treatment using imaging is based on whether tumors shrink, stay the same, or get bigger. The complete disappearance of all target lesions is referred to be a CR. A PR is characterized by a minimum 30% reduction in the tumor's greatest axis size. The size of the target disease increased by at least 20% as the disease progressed.^10^

#### CTCAE V.5

CTCAE V.5 is a scale used to define any abnormal clinical finding associated with the use of cancer therapy.^11^

#### Advanced HNC

Advanced HNC is the HNC that is unlikely to be cured or controlled with treatment.^12^

#### QoL

The QLQ-C30 and QLQ-HN-43 are made up of multi-item scales as well as single-item measures. Five functional scales, three symptom scales, a global health status/QoL scale, and six single items are among them. The scales and single-item measures all have a score range of 0-100. Thus, a high score on a functional scale represents a healthy level of functioning; a high score on the global health status/QoL represents a high QoL; a high score on a symptom scale represents a high level of symptomatology. The score difference after treatment shows no change for all QoL parameters if it is between –10 and 10 points. For physical and global functional scores, improvement is when the difference is more than 10 points. For a symptom score, improvement is when the difference is less than –10 points.^13,14^

#### Hypofractionated RT

Hypofractionated RT is the radiation treatment in which the total dose of radiation is divided into large doses and treatments are given once a day or less often. In this study, regimens were 30 Gy in 10 fractions, one fraction per day and 5 times per week and completed in 2 weeks., and 20 Gy in five fractions, one fraction per day and 5 times per week.

#### QUAD-SHOT RT

Quad-shot RT is a cyclical hypofractionated palliative RT regimen consisting of 14 Gy in four fractions, given twice a day and at least 6 hours apart, for 2 consecutive days (Saturday and Sunday). This radiation treatment plan was repeated at four weekly intervals for a further two courses which mean a total of 44.4 Gy.^15^

#### RT Techniques

We used a linear accelerator (LINAC) machine for treatment. Patients were simulated supine in CT scan after providing informed consent on the basis of their respective protocols for their primary site. All patients were treated with three-dimensional conformal RT and a dose was prescribed to the midplane at the central axis. To preserve as much normal mucosa as possible, fields are formed using multileaf collimators.

### Statistical Analysis

The supervisors and, finally, the principal investigator have reviewed the data for completeness and consistency. To minimize data entry errors, the data were double-entered into Epidata 4.6 and then exported to SPSS version 26 (IBM Corp, Armonk, NY) for analysis after cleaning and editing. The analyses included all patients who had finished their planned course of RT. Descriptive statistics such as percentage and frequency were used for all treatment end points. Overall survival estimation is done with the Kaplan-Meier survival curve.

The best level of symptom control and QoL achieved after treatment was coded compared with pretreatment levels. We used four categorical scores for response assessment of index symptoms; none (CR), mild to moderate (PR), no change (stable), and severe (progressive). QoL data were classified as worse than, no change from, or better than pretreatment levels. The worst grade of post-treatment toxicities observed after the start of the first course of RT was coded.

## RESULTS

### Sociodemographic Characteristics

From January 2022 to January 2023, about 316 patients were registered; 182 patients were booked for curative treatment using either chemoradiation alone or induction chemotherapy followed by chemoradiation. About 103 patients were booked for palliative care using radiation, chemotherapy, or both, depending on their symptoms. About 31 patients were booked for the best supportive care. However, only a small number of patients received radical treatment because of the long waiting time. The treating oncologist chose patients who required rapid symptom relief. Six patients who experienced tumor progression or worsening of symptoms after chemotherapy were included in the study. Seven patients who underwent surgery and experienced tumor progression while awaiting RT were included. A total of 52 patients fulfilled the inclusion criteria during study period. However, 10 patients did not complete the preplanned RT because of machine failure during treatment and 15 patients were not started treatment until the time of final data analysis. Two patients refused to participate in the study. Finally, 25 patients completed treatment and were eligible for outcome assessment. Biopsy was used to confirm the diagnosis in 22 patients and fine needle aspiration cytology (FNAC) was used in three cases.

The median age of diagnosis was 55 years (the minimum being 20 and the maximum being 84 years). Fourteen patients (56%) were female. Six patients had related comorbidities, three of which were diabetes and three of which were hypertension.

Regards to functional status, 21 (84%) patients were Eastern Cooperative Oncology Group (ECOG) 1 and four patients were ECOG 2. The commonest anatomic site was paranasal sinus, five (20%), followed by nasopharynx and thyroid cancers, four (16%) each. The most common histologic type was squamous cell carcinoma (SCC), nine (36%), followed by adenoid cystic carcinoma, four (16%). The majority of the patients were American Joint Committee on Cancer stage IVB at the time of treatment initiation, 14 (56%; Table 1).

**TABLE 1:**
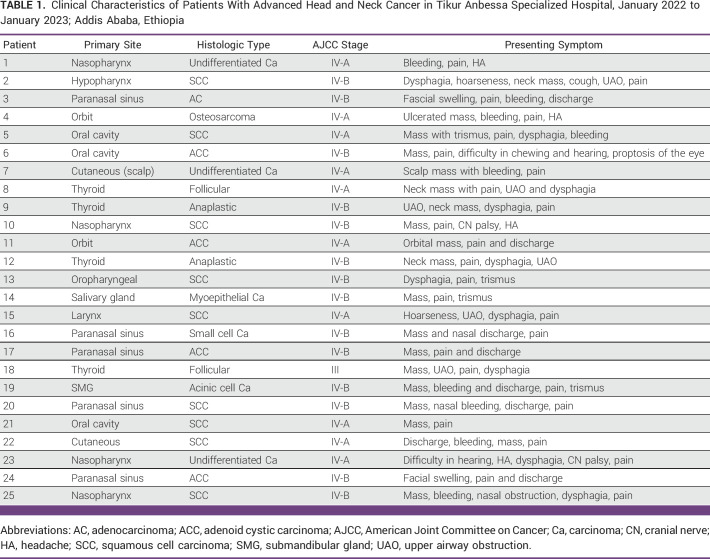
Clinical Characteristics of Patients With Advanced Head and Neck Cancer in Tikur Anbessa Specialized Hospital, January 2022 to January 2023; Addis Ababa, Ethiopia

In terms of treatment, the quad-shot (the 44.4 Gy in 12 fraction) and 30 Gy in 10 fractions each account for nine (36%) of the patients. Eight patients underwent chemotherapy, with six receiving it before RT and two receiving it after treatment had been completed for 1 month. Seven (28%) patients underwent curative surgical resection before initiation of RT.

### Treatment Response

Regarding symptom relief, all patients had pain at the tumor site at presentation. Of them, 13 (52%) had a subjective CR to treatment, while 12 had a subjective PR but still needed additional analgesics to relieve the pain. Thirteen patients presented with active bleeding and 11 (44%) of them attained complete hemostasis. Three of the 10 patients who reported dysphagia had a CR, while seven others had a PR. Six patients exhibited symptoms of impending upper airway obstruction; one had a complete recovery, five had partial recovery, and at least more surgical treatments (tracheostomy) were not required in any of the five patients. We assessed the objective response rate (ORR) using RECIST criteria, and none of the patients attained a CR. However, at the end of treatment, 16 (64%) patients had a PR and nine patients had stable disease (Table 2).

**TABLE 2:**
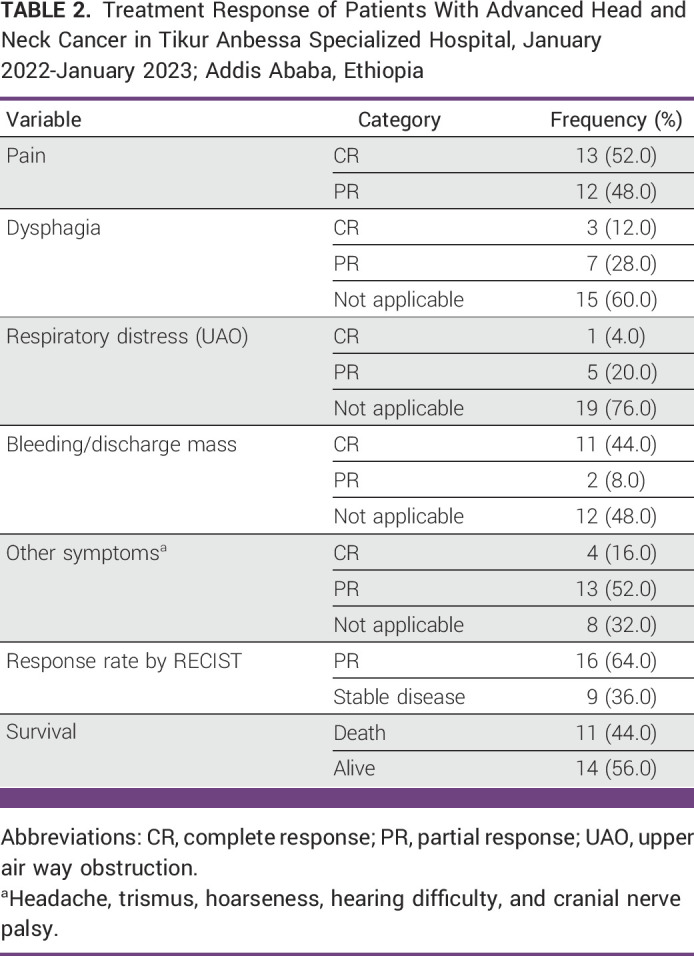
Treatment Response of Patients With Advanced Head and Neck Cancer in Tikur Anbessa Specialized Hospital, January 2022-January 2023; Addis Ababa, Ethiopia

**FIG 1:**
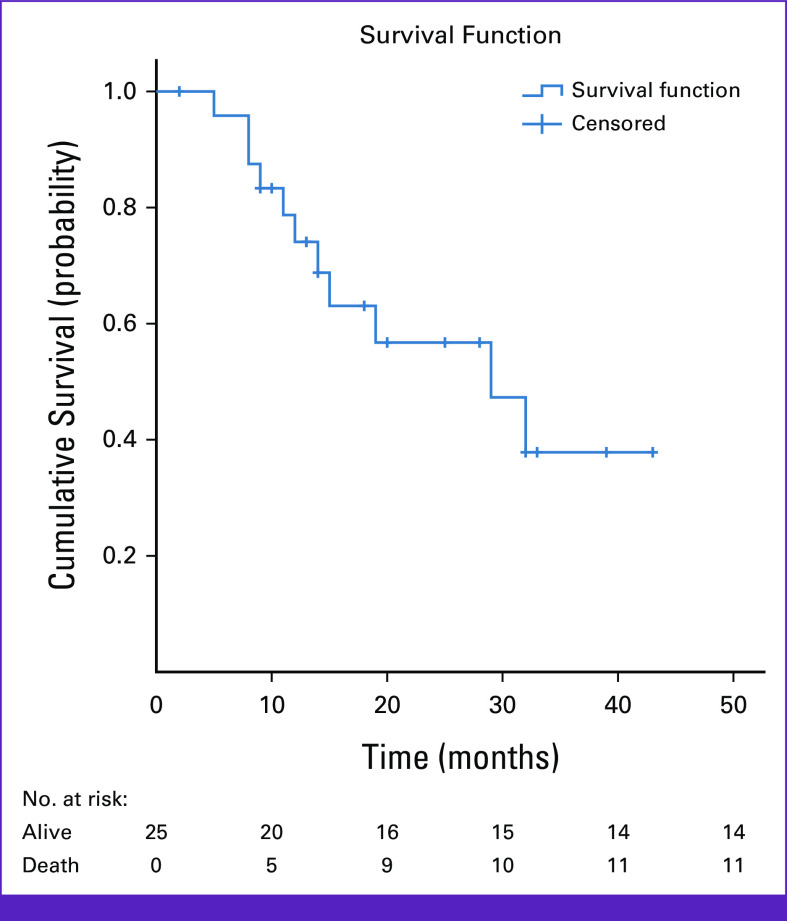
Kaplan-Meier overall survival curve for patients with advanced head and neck cancer treated with palliative hypofractionated RT regimens in Tikur Anbessa Specialized Hospital, January 2022-January 2023, Addis Ababa, Ethiopia. RT, radiotherapy.

**FIG 2:**
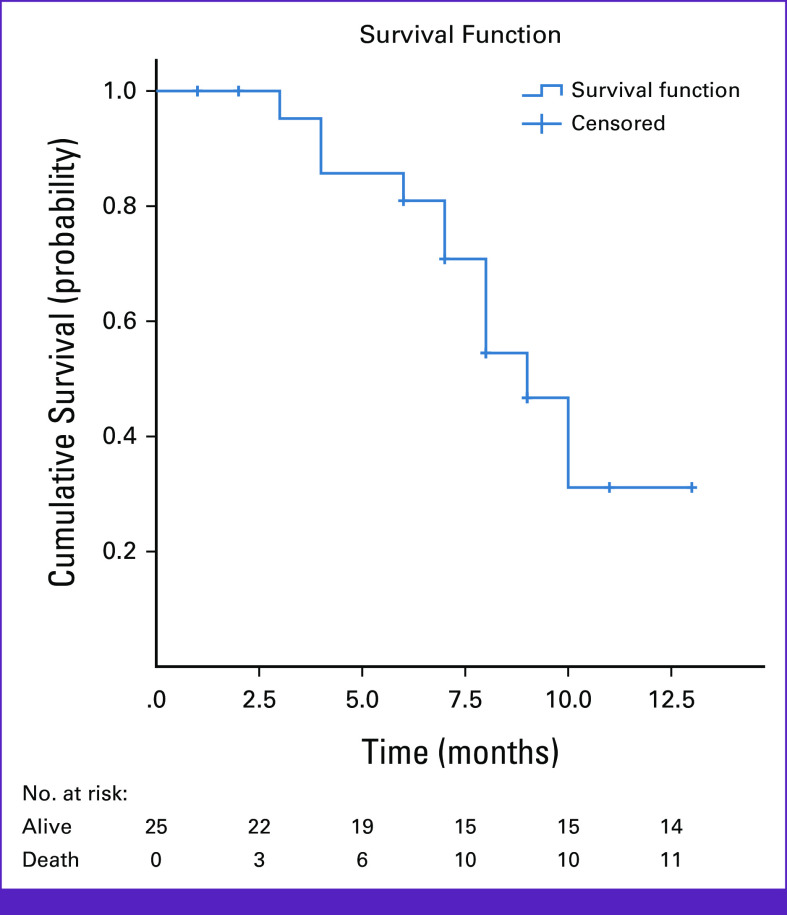
Kaplan-Meier survival curve after the completion of RT for patients with advanced head and neck cancer treated with palliative hypofractionated RT regimens in Tikur Anbessa Specialized Hospital, January 2022-January 2023, Addis Ababa, Ethiopia. RT, radiotherapy.

At the end of the study period, 11 (44%) patients died, with an overall median survival of 29 months (95% CI, 11.8 to 46.2; Fig 1). The median survival after completion of radiation, however, was 9 months (95% CI, 7.2 to 10.8), which is calculated from the last date of RT (Fig 2). The mean follow-up time after radiation was approximately 7 months (6.7 months with standard deviation of 3.1; Fig 2).

### Treatment Toxicities

Grade 2 dermatitis, which was observed in 13 (52%) patients, was the most frequent treatment-related adverse effect. It was followed by grade 2 xerostomia and grade 2 mucositis in 11 (44%) patients. There were no documented treatment-related grade 3 or higher toxicities, except for one patient who had grade 3 xerostomia (Table 3).

**TABLE 3:**
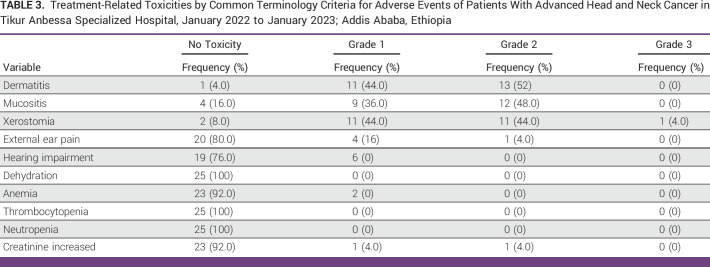
Treatment-Related Toxicities by Common Terminology Criteria for Adverse Events of Patients With Advanced Head and Neck Cancer in Tikur Anbessa Specialized Hospital, January 2022 to January 2023; Addis Ababa, Ethiopia

### QoL

In terms of QoL, we selected factors in QLQ-30 and QLQ.HN-43 that are associated with symptoms of HNC. Hence, all chosen physical scores were improved in more than 12 (48%) of the irradiated patients. More than 20 patients (80%) showed improvement in their global functional score, whereas just one patient had an overall global functional score decline. In terms of symptom score, 16 (64%) patients improved with their symptoms of swallowing and chewing problems. However, six (24%) and three (12%) patients had worsened symptoms of dry mouth and taste difficulty, respectively (Table 4).

**TABLE 4:**
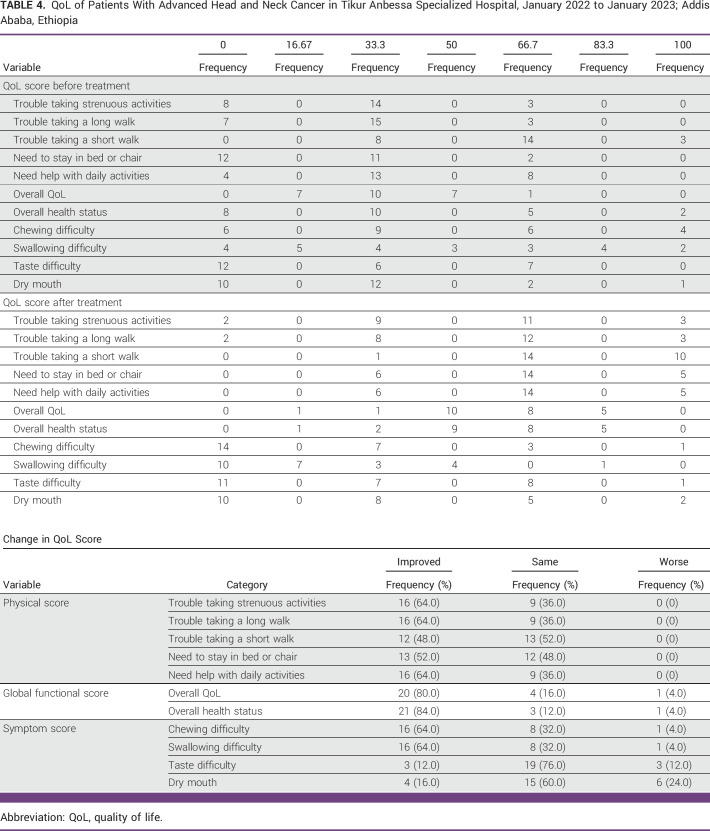
QoL of Patients With Advanced Head and Neck Cancer in Tikur Anbessa Specialized Hospital, January 2022 to January 2023; Addis Ababa, Ethiopia

When we compare the different RT regimens and outcomes showed that quad-shots had better symptom response, ORR, and QoL scores, but more mucositis. 20 Gy in five fractions had less dermatitis. Survival was similar among groups (Data Supplement, Tables S1-S3).

## DISCUSSION

The primary goal of palliative care is to reduce symptoms and increase the QoL, particularly in cases with advanced-stage presentation and in resource-limited settings such as Ethiopia. As a result, nearly all the cases in our study were locally advanced and stage IV diseases with overwhelming symptoms such as pain and bleeding from the tumor, as well as dramatically reduced QoL. Even for patients who are candidates for curative treatment, acute symptom relief should be a priority because of a lack of timely treatment administration.

In our study, complete pain palliation was the most common treatment outcome seen in our patients (52%). Successful pain control was also seen in an Australian study with a regimen of 30 Gy in five fractions.^16^ In our study, 11 of 13 patients who first presented with bleeding or discharge had completely resolved their symptoms. In our investigation, other presenting symptoms, including dysphagia, trismus, and hoarseness, are also significantly improved. This is demonstrated across all hypofractionated regimens and tumor types, but the relative merits of each regimen cannot be evaluated because of the small number of patients. A study conducted at the Mayo Clinic demonstrated the effectiveness of the quad-shot regimen for symptom palliation.^17^ In Indian patients, a greater dose of palliative regimen (40 Gy in 10 fractions) was also found to be effective but at the cost of increased toxicities.^18^ In addition, other existing regimens, such as 28 Gy in eight fractions, are effective in symptom management.^19^ The main difference between our study and other studies is that we included all histologic subtypes in which symptom improvement is found, whereas most other studies only addressed SCC.

Palliative radiation improves the rate of the objective tumor response in addition to reducing symptoms. In our study, at the end of fourth week of RT, 16 (64%) patients had a PR by RECIST criteria and nine (36%) patients had a stable tumor size. This PR has led to the improvements of six (24%) patients who presented with symptoms of airway obstruction because of mass effect. However, according to the RECIST criteria, none of the patients experienced a CR, which is also not predicted, considering the dose given to our patients. None of the patients had disease progression at 1 month. In India, there was almost a similar ORR along with significant symptom relief.^19^ In contrast to our findings, research conducted in the Netherlands used 50 Gy in 16 fractions for SCC and 45% of patients achieved a CR. This could be because all patients had a considerably greater dosage of radiation with equivalent dose in 2 Gy fractions (EQD2) for tumors about 54.69 Gy^20^ compared with our patient's maximal dose with a quad-shot regimen for nine patients, where the EQD2 is 50.69 Gy. In a long retrospective follow-up study, a CR rate of up to 30% was observed with 24 Gy in three fractions.^9^

Another significant end point evaluation performed among our patients was the assessment of the specified components in both general and head and neck–specific QoL measures. For 48% of patients, their QoL improved in one parameter of the physical scores. Moreover, 64% of patients showed improvement in three parameters. The global functional score improved in at least 80% of patients. For the symptom score, chewing and swallowing difficulty improved for 64% of patients in each category. A significant improvement in patient-reported QoL was shown in a phase two study from Canada using 25 Gy in five fractions.^12^ In a similar study in Australia, almost 44% of 25 patients improved their QoL with a quad-shot regimen.^15^ Other available choices, such as 30 Gy in five fractions^16^ and 40 Gy in 10 fractions,^20^ are also effective for improving QoL. However, in our study, on the basis of QoL symptom scores, six (24%) and three (12%) patients exhibited increased symptoms of dry mouth and taste difficulty, respectively. This could be explained by the xerostomia and mucositis observed after treatment. Our investigation along with the aforementioned evidences demonstrated that hypofractionated regimens improve QoL.

Although overall survival is the gold standard end point in cancer, the primary goal of cancer treatment in cases with an incurable disease is not to improve survival. Health-related QoL, however, is a surrogate marker for overall survival. After completion of RT with a mean follow-up of 7 months, the overall mortality rate in this study was 44%, with a median survival period after radiation of 9 months. This is comparable with a similar study done in Australian patients, where the median time to death was 6.1 months.^16^ However, the Netherlands data revealed that the median survival period was 17 months, which was longer than that of our patients.^16^ This could be because this study included stage I/II patients, a higher radiation dose, and all cases were SCC. For a certain group of patients, it makes sense to implement a higher-dose palliative regimen, which may increase survival along with symptoms and local tumor control.

In this study, there were no patients with dermatitis or mucositis of grade 3 or higher. These results coincide with those that have been published in previous studies as well as the AIIMS study.^15,21,22^ However, in the Dutch Christie scheme, the hypo trial, and Christian Medical College Vellore Experience of different hypofractionated radiation studies for patients with comparable conditions, there were 65%, 26%, and 18% with grade three mucositis and 45%, 11%, and 3% with grade three dermatitis, respectively.^16,18,23^ This might be because patients in our study often received lower cumulative dosages than those used in earlier trials, which used larger cumulative doses. Except for one patient who developed grade 3 xerostomia during this brief period of follow-up, 88% of patients experienced xerostomia in grade 1 or 2. Poor functional status, anemia, and stage IVB at presentation might have attributed to the patient's grade 3 toxicity. The findings from this research are consistent with those of the study by Corry et al,^15^ which revealed grade 1 and 2 salivary gland damage in 67% of patients, with the majority of these patients receiving full courses of quad-shot regimen. As a result, the lack of severe toxicities in our patients makes it more practical and acceptable in the setting of palliative care without compromising symptom control and patient QOL.

We compared outcomes among regimens without robust statistics because of small sample size. Quad-shots had higher CR, ORR, and QoL scores, but more mucositis. Twenty Gy in five fractions had less dermatitis. This may be due to different biologically effective dose. Survival was similar among groups. More patients are needed to confirm more effective and the safest regimen.

In conclusion, all palliative hypofractionated regimens used were effective in terms of symptom control, tumor response rate, and QoL, and were well tolerated. This makes it appropriate for our setup because the majority of patients require palliation. We recommend implementing these hypofractionated regimens for appropriately selected patients.

The strength of the study is the prospective design of the study and the fact that it was conducted in the most prominent and high-volume cancer center in Ethiopia. To determine whether the treatment was appropriate, all treatment outcomes were also evaluated.

We were limited in our ability to include a large number of patients to look for determinant factors of patient outcome for subgroup analysis, such as histologic subtypes and types of regimens.

## Data Availability

The data used for this research will be available upon request.
